# *Bacillus anthracis*-Like Bacteria and Other *B. cereus* Group Members in a Microbial Community Within the International Space Station: A Challenge for Rapid and Easy Molecular Detection of Virulent *B. anthracis*

**DOI:** 10.1371/journal.pone.0098871

**Published:** 2014-06-19

**Authors:** Sandra P. van Tongeren, Hendrik I. J. Roest, John E. Degener, Hermie J. M. Harmsen

**Affiliations:** 1 Department of Medical Microbiology, University Medical Center Groningen, University of Groningen, Groningen, The Netherlands; 2 Department of Bacteriology & TSEs, Central Veterinary Institute (CVI), part of Wageningen UR, Lelystad, The Netherlands; Rockefeller University, United States of America

## Abstract

For some microbial species, such as *Bacillus anthracis*, the etiologic agent of the disease anthrax, correct detection and identification by molecular methods can be problematic. The detection of virulent *B. anthracis* is challenging due to multiple virulence markers that need to be present in order for *B. anthracis* to be virulent and its close relationship to *Bacillus cereus* and other members of the *B. cereus* group. This is especially the case in environments where build-up of *Bacillus* spores can occur and several representatives of the *B. cereus* group may be present, which increases the chance for false-positives. In this study we show the presence of *B. anthracis*-like bacteria and other members of the *B. cereus* group in a microbial community within the human environment of the *International Space Station* and their preliminary identification by using conventional culturing as well as molecular techniques including 16S rDNA sequencing, PCR and real-time PCR. Our study shows that when monitoring the microbial hygiene in a given human environment, health risk assessment is troublesome in the case of virulent *B. anthracis*, especially if this should be done with rapid, easy to apply and on-site molecular methods.

## Introduction

Monitoring of microbial hygiene can give an early warning sign for the presence of potential pathogens in an environment so preventive countermeasures can be taken to minimise health risks. To this purpose, microbial hygiene is monitored and controlled at various locations such as hospital wards, potable water distribution systems or air-conditioning systems of modern buildings but also in spacecraft such as the *International Space Station* (ISS). To secure the health and wellbeing of the crew, characterisation and understanding of the microbial contamination of the ISS is essential [24]. Especially at remote locations such as the ISS, rapid and easy to use methods to monitor microbial hygiene are preferred, which can be used on-site without the need for technical personnel or specialised laboratories. Detection and identification of microbes by conventional culturing is laborious, time-consuming, requires specialist expertise and many species are difficult to culture or uncultivable. In addition, culturing of hazardous microbes such as potential pathogens or so-called technophiles [33] is often undesirable. In this respect, molecular detection methods circumvent many of the disadvantages encountered by conventional culturing techniques [35]. However, for some species, such as *Bacillus anthracis*, the etiologic agent of the disease anthrax, rapid and easy on-site molecular detection can be problematic.

The ISS is a complex environment with a regulated atmosphere which forms a unique microbial ecological niche that encompasses several spaceflight related parameters such as microgravity, radiation or the concept of closed environment [14]. The secondly most common identified bacterial species in the ISS environment are spore-formers of the genus *Bacillus* (31.7%) [24]. *Bacillus* species generally are aerobic endospore-forming bacteria that are common in nature. More specifically, the *Bacillus cereus* group comprises six members: *Bacillus cereus*, *Bacillus anthracis*, *Bacillus thuringiensis, Bacillus mycoides*, *Bacillus pseudomycoides*, and *Bacillus weihenstephanensis*. *B. cereus*, *B. anthracis*, and *B. thuringiensis* can be considered to be pathovars of a single species and it has been suggested that *B. anthracis* is a monophyletic clone derived from the *B. cereus* and *B. thuringiensis* clade [21], [25], [28], [29]. Whilst *B. cereus* is an opportunistic pathogen that also causes food borne illness, *B. thuringiensis* is an insect pathogen. *B. anthracis* is an obligate pathogenic species that causes the zoönosis anthrax [5], [22]. The disease primarily occurs in herbivores, and is usually transmitted to humans by direct or indirect contact with contaminated animals. Two plasmids are essential for the virulence of *B. anthracis*, pXO1 and pXO2 [5], [22]. Plasmid pXO1 harbours genes for the anthrax toxin complex proteins edema factor (*cya*), lethal factor (*lef)* and protective antigen (*pag*A) [26] and pXO2 carries genes required for the biosynthesis of an antiphagocytic poly-γ-D-glutamic acid capsule (*capBCA*). Rapid detection of virulent *B. anthracis* in the environment is essential since the infection spreads rapidly and has a high mortality rate, but is challenging due to its close relationship to other members of the *B. cereus* group [16], [29].

Strains of the *B. cereus* group were isolated from the interior of the ISS for the purpose of investigating the bacterial microbiota, as part of a Microgravity Applications Programme (MAP) of the European Space Agency and in particular the ‘SAMPLE’ experiments [33], and were characterised using both phenotypic and molecular methods. The aim of this study was to describe the difficulties encountered for rapid and easy on-site molecular detection of virulent *B. anthracis* in microbial communities which was demonstrated with the discovery and preliminary classification of *B. anthracis*-like isolates and other members of the *B. cereus* group found to be present in the same human environment of the ISS.

## Materials and Methods

### Sample Collection

Samples were collected from various locations of the interior of the Russian *Zvezda* Service Module (DOS-8) of the ISS during several spaceflight missions by mainly using a sampling system consisting of a Swab Rinse Kit (927C SRK) tube without medium containing a Dacron applicator (Copan Italia S. p. A. Diagnostics Inc., Brescia, Italy). Swabs were pre-moistened with 55 µl sterile clinical grade 0.9% physiological salt solution (B. Braun Melsungen AG, Melsungen, Germany) using DNA-free laboratory techniques. The samples were stored and transported to the microbiology laboratory on Earth at the University Medical Center Groningen. The samples were suspended in Fastidious Broth (FB; Mediaproducts BV, Groningen, The Netherlands) and incubated for 1–2 days at 37°C, after which the suspension was plated on Blood Agar (Mediaproducts BV) and incubated for another 1–2 days at 37°C. Pure cultures of isolates were maintained in Microbank tubes (Pro-Lab Diagnostics) at −80°C and further cultivated overnight on Blood Agar plates at 37°C for various analyses.

### Preparation of Template DNA for PCR and Sequencing

Bacterial cells were inoculated into 9 ml Brain Heart Infusion (Mediaproducts BV) and grown overnight at 37°C. From this suspension, template DNA was prepared by using either whole bacterial cells without extracting DNA, or extracting DNA based on the method of Boom et al. [3]. Briefly, in the latter case, 0.1 ml bacterial cell suspension was added to 600 µl mixture of L_6_ and Celite 545 (Acros Organics, Geel, Belgium), and put at ambient temperature for 10 min. The mixture was centrifuged in an Eppendorf microcentrifuge for 10 s at 13,400×g. The pellet was washed with 70% ethanol (v/v) and subsequently acetone and centrifuged for 10 s at 13,400×g after each washing step. The acetone was disposed of after which the pellet was left to dry for 10 min at 56°C. The pellet was dissolved in TE buffer (10 mM Tris-HCl, 1 mM EDTA, pH 8.0) during 10 min at 56°C.

### 16S rRNA Gene Sequencing

Conserved eubacterial primers based on Hiraishi et al. [10] were used to amplify 16S rRNA genes by PCR (Table 1). To enable direct cycle sequencing of the PCR product after amplification, a short T7 sequence was attached to the 5′ side of the forward primer and a UP sequence to the 5′ side of the reverse primer (Table 1). Oligonucleotide primers were obtained from Biolegio (Nijmegen, The Netherlands). Template DNA was prepared as described above using whole bacterial cells. All PCR reactions were performed in a total volume of 50 µl consisting of 1 or 5 µl of template DNA, 0.5 U *Super TAQ* HC (HT Biotechnology Ltd., Cambridge, England), PCR Buffer (HT Biotechnology Ltd.), 200 µM of each dNTP (Fermentas Int. Inc., Burlington, Canada) and 100 nM of each primer. Amplification was carried out on an Omnigene TR3 CM220 Temperature Cycler (Hybaid Ltd., London, UK) with a profile of 94°C for 5 min, followed by 50 cycles of 94°C for 30 s, 58°C for 75 s and 72°C for 1.5 min and finally one cycle of 72°C for 5 min. To confirm amplification of PCR products of the proper size, electrophoresis was performed through an ethidium bromide stained 1.0% (w/v) agarose gel (Eurogentec S.A., Seraing, Belgium) in TBE buffer (Invitrogen Ltd., Paisley, UK). PCR products were bidirectionally sequenced (BaseClear BV, Leiden, The Netherlands) by using T7, UP and internal primers with proprietary in-house protocols.

**Table 1:** PCR oligonucleotide primers used in this study.

Target gene	Primer	Sequence (5′–3′)	References
*gyrB*	BC1	ATTGGTGACACCGATCAAACA	Yamada et al. [34]
	BC2r	TCATACGTATGGATGTTATTC	Yamada et al. [34]
*gyrB*	BA1	AATCGTAATATTAAACTGACG	Yamada et al. [34]
	BA2r	CCTTCATACGTGTGAATGTTG	Yamada et al. [34]
*gyrB*	BT1	ATCGGTGATACAGATAAGACT	Yamada et al. [34]
	BT2r	CCTTCATACGTATGAATAATTTTT	Adapted from Yamada et al. [34]
16S rRNA	forward	**ACCTAATACGACTCACTATAGGG** AGAGTTTGATCCTGGCTCAG	(R. Rozeboom and P. Terpstra, personalcommunication); Hiraishi et al. [10]
16S rRNA	reverse	**ATTGTAAAACGACGGCCAGT** GGTTACCTTGTTACGACTT	(R. Rozeboom and P. Terpstra, personalcommunication); Hiraishi et al. [10]
BA_5345	BA chrom F	TGCATTACATACCCCAAAGGTACA	based on Antwerpen et al. [1]
	BA chrom R	TTTGTCACAAATGAGCAAAGGTTT	based on Antwerpen et al. [1]
	BA chrom probe	**6-FAM**-CCACAGCGGCTAAAGAGACCAGTAACCC-**BHQ-1**	based on Antwerpen et al. [1]
*lef*	Lef F	TTGATATAAATGAAAGGCCTGCATT	based on Hadjinicolaou et al, 2009 [8]
	Lef R	TTCCAGACCGATGTTTCTTTGTAA	based on Hadjinicolaou et al, 2009 [8]
	Lef probe	**CY3**-AGCGTTTGAAATGGAGAATCCAATTATCACCAG-**BHQ-2**	based on Hadjinicolaou et al, 2009 [8]
*capC*	CapC F	CGTATGGTGTTTCAAGATTCATGAT	based on Hadjinicolaou et al, 2009 [8]
	CapC R	CTCAAATGGCATAACAGGATAACAA	based on Hadjinicolaou et al, 2009 [8]
	CapC probe	**CY5**-TGGCCGTAGAAAATTTGCGGCAAC-**BHQ-2**	based on Hadjinicolaou et al, 2009 [8]

Bold: T7 and UP extension, respectively.

Abbreviations: FAM, carboxyfluorescein; CY, cyanine; BHQ, Black Hole Quencher.

### PCR of *gyrB*

A *Bacillus* species-specific chromosomal region was amplified with a PCR assay based on Yamada et al. [34]. Bacterial primers were used to amplify a fragment of the g*yrB* gene of *B. cereus* (BC1 and BC2r, 365 bp), *B. anthracis* (BA1 and BA2r, 245 bp) and *B. thuringiensis* (BT1 and BT2r, 368 bp) (Table 1). Oligonucleotide primers BC1, BC2r, BA1 and BA2r were obtained from Eurogentec S.A. and BT1 and BT2r from Biolegio. Template DNA was prepared as described above using whole bacterial cells or DNA solution in duplicate (four-fold, in total). All PCR reactions were performed in a total volume of 25 µl containing 1 or 5 µl of template DNA, 0.25 U *Super TAQ* HC (HT Biotechnology Ltd.), PCR Buffer (HT Biotechnology Ltd.), 200 µM of each dNTP (Fermentas Int. Inc.), 200 nM of each primer and 1 mM MgCl_2_. Amplification was carried out on a PTC-225 DNA Engine Tetrad (MJ Research Inc., Massachusetts, USA) as described previously [34]. To confirm amplification of PCR products of the proper sizes, electrophoresis was performed through an ethidium bromide stained 1.0% (w/v) agarose gel (Eurogentec S.A.) in TBE buffer (Invitrogen Ltd.).

### Expert Confirmation Analysis

Expert confirmation analysis was conducted on the isolates by the Dutch national veterinary reference laboratory (CVI, WUR, Lelystad, The Netherlands) which encompassed microscopic evaluation of a Gram-stained preparation, evaluation of β-haemolysis, γ-phage susceptibility testing and an in-house *B. anthracis* PCR. γ-phage susceptibility is tested for by the capacity of the phage to lyse the culture.

Primers and probes of the PCR specific for *B. anthracis* were developed with Primer Express 2.0 (Applied Biosystems, Carlsbad, CA, USA) and based on Antwerpen et al. 2008 [1] and Hadjinicolaou et al. 2009 [8] (Table 1). Primers were used to amplify a specific *B. anthracis* chromosomal fragment with locus tag BA_5345 encoding for a hypothetical protein (BA chrom F and R, 78 bp), the *lef* gene on plasmid pXO1 (Lef F and R, 128 bp) and the *capC* gene on plasmid pXO2 (CapC F and R, 119 bp). All PCR reactions were performed in a total volume of 20 µl, consisting of 5 µl of template DNA (prepared according to Boom et al. [3]), 14 µl of amplification mixture containing 400 nM of primers (Eurogentec S. A.) and 200 nM of probes (Eurogentec S. A.), PCR reaction buffer (PerfeCTa MultiPlex qPCR SuperMix (UNG, Low ROX), Quanta BioSciences Inc., MD, USA) and water and 1 µl of inhibition control (IC). The PerfeCTa Multiplex qPCR Supermix reagents contained uracil-N-glycosylase (UNG) to prevent carry-over contamination. The IC was constructed for amplification with the primers of the chromosomal fragment and specific detection with a dedicated probe (5′-3′: **Yakima Yellow**-CGTGGATCACCCACAAGAGCCCAC-**Black Hole Quencher-1**). PCR was performed on a 7500 Fast Real-Time PCR System (Applied Biosystems) with a profile of 45°C for 5 min to activate UNG and 95°C for 60 s, followed by 40 cycles of 95°C for 10 s and 60°C for 30 s. Positive (DNA from *B. anthracis* strain N85) and negative controls (water) were included. Results were generated with 7500 Fast System Software (Applied Biosystems).

### Biochemical and Phenotypic Characterisation

Biochemical and phenotypic characterisation was performed on pure cultures by making use of the API system (bioMérieux Inc., Marcy l’Etoile, France) according to the manufacturer’s instructions. The API 20 E tests were used to test for arginine dihydrolase, indole production, acetoin production (VP), gelatinase and nitrate reduction. Inconclusive reactions for acetoin production and nitrate reduction were confirmed with standard culture-based techniques [7]. Anaerobic acid production from D-arabinose, glycerol, glycogen, inulin, D-mannitol, salicin and D-trehalose was determined using the API 50 CHB system (bioMérieux Inc.). Supplemental tests were performed by using the VITEK 2 Systems *Bacillus* identification card (BCL) (bioMérieux Inc.) and standard culture-based techniques [7]. Anaerobic growth was determined by cultivation on Brucella Blood Agar (Mediaproducts BV) under a nitrogen atmosphere. Capsule production was tested for by using M’Fadyean polychrome methylene blue and Indian ink stain, on smears of cells that were cultivated on nutrient agar containing 0.8% sodium bicarbonate under a 5% CO_2_ atmosphere.

### Data Analysis

Nucleotide sequences were aligned by using the BioEdit Sequence Alignment Editor version 7.0.8.0 [9]. Sequence analysis was conducted on determined sequences by searches of the online BLASTN [36] and RDP10 [4] databases.

## Results

Eleven *B. cereus* group isolates were selected based on morphology and Gram stain from a collection of strains isolated from the interior of the Russian *Zvezda* Service Module of the ISS. These strains were isolated from various sites of the human living quarters, including walls, tables and toilet areas. All S1 isolates were isolated from a first flight and the S2 isolates from a second flight one year later (Table 2). S1-R3O1-FB and S2-R3O1-FB-BA1 were isolated from the same wall panel, but during two consecutive flights one year apart.

**Table 2:** Overview of isolate characteristics using several methodologies.

Character	*B. cereus*	*B. anthracis*	*B. thuringiensis*	*B. mycoides*	S1-R1P1-FB	S1-R4D1-FB	S1-R6TC1	S1-R3O1-FB	S2-R3O1-FB-BA1	S2-R4W1-FB-BA1	S1-R1J2-FB	S1-R2T1-FB	S1-R4H1-FB	S2-R3J1-FB-BA1	S1-R5C1-FB
16S rDNA analysisby online database searches	*B. cereus* group														
					*B. cereus*					*B. weihenstephanensis*	Inconclusive				
PCR of *gyrB*:															
* B. cereus*	+				+	+	+	+	+	−	−	−	−	−	−
* B. anthracis*		+			−	−	−	−	−	(+)	+	+	+	+	+
* B. thuringiensis*			+		−	−	−	−	−	−	−	−	−	−	−
Colony morphology					¤	¤	¤	¤	¤	Δ	X	X	X	X	¤
β-haemolysis	+	−/(+)	+	−/(+)	+	+	+	(+)	(+)	+	+	+	+	+	+
Motility	+	−	+	−	+	(+)	+	(+)	(+)	+	+	+	+	+	+
Parasporal crystals	−	−	+	−	−	−	−	−	−	−	−	−	−	−	−
Acetoin production	+	+	+	+	+	−	+	+	−	+	+	+	+	+	+
Casein hydrolysis	+	+	+	+	−	+	−	−	−	+	+	+	+	+	+
Penicillin susceptibility	R	S/(R)	R		R	R	R	R	R	R	R	R	R	R	R
Capsule production	−	+/−	−	−	−	−	−	−	−	−	−	−	−	−	−
Gamma phage susceptibility*	−	+	−	−	−	−	−	−	−	−	−	−	−	−	(+)
Arginine dihydrolase	v	−	+	V	+	+	+	+	+	−	+	+	+	+	+
Acid from:															
Glycerol	+	−	+	+	−	−	−	−	−	−	−	−	−	−	−
Salicin	+	−	(+)	(+)	+	+	+	+	+	+	(+)	−	(+)	−	(+)
PEA		−			+	+	+	+	+	(+)	+	+	+	+	+
VITEK 2 Systems BCL card															
L-aspartate arylamidase	ND	ND	ND	ND	−	−	−	−	−	−	−	−	−	−	+
Leucine arylamidase	ND	ND	ND	ND	+	+	+	(+)	+	+	(+)	(+)	(+)	+	+
Alanine arylamidase	ND	ND	ND	ND	+	+	+	+	+	+	−	−	−	−	−
Tyrosine arylamidase	ND	ND	ND	ND	+	+	(+)	+	+	+	+	+	+	+	+
Cyclodextrine	ND	ND	ND	ND	−	−	−	−	+	−	−	−	−	(+)	−
Glycine arylamidase	ND	ND	ND	ND	−	−	−	−	−	−	+	−	(−)	−	(−)
D-mannose	ND	ND	ND	ND	−	−	−	−	−	+	−	−	−	−	−
N-acetyl-D-glucosamine	ND	ND	ND	ND	+	+	+	+	+	+	+	−	+	+	(−)
β-glucosidase	ND	ND	ND	ND	+	+	+	+	−	+	−	−	−	−	−
Pyruvate	ND	ND	ND	ND	(+)	+	+	(+)	+	(+)	(+)	+	+	+	+
α-glucosidase	ND	ND	ND	ND	−	−	−	(+)	+	−	−	−	(−)	(−)	−
Growth in 6.5% NaCl	ND	ND	ND	ND	+	+	+	+	+	−	+	+	+	+	+
Kanamycin susceptibility	ND	ND	ND	ND	R	R	R	R	S	S	R	R	R	R	R
Tetrazolium red	ND	ND	ND	ND	−	+	(−)	+	−	−	−	+	(−)	+	−

Abbreviations: +, positive; v, variable; (+) or (−), weak reaction; −, negative; S, susceptible; R, resistant; ND, not determined. Symbols for colony morphology indicate similarity and differences. All data were obtained in this study, except data for *B. cereus*, *B. anthracis*, *B. thuringiensis* and *B. mycoides* that were compiled [21], [22].

*Gamma phage susceptibility is not truly specific for *B. anthracis* [32].

### 16S rDNA Sequence Analysis

16S rDNA sequence analysis results of searches of the online databases are shown in Table 2. Identification to the species level of isolates S1-R1J2-FB, S1-R2T1-FB, S1-R4H1-FB, S2-R3J1-FB-BA1 and S1-R5C1-FB (referred to as the BA isolates, hereafter) was inconclusive. Subsequently, signature sequences of the 16S rDNA of isolates in this study were compared with those of the *B. cereus* group types according to the scheme of Sacchi et al [31], as shown in Table 3. Isolates S1-R1P1-FB, S1-R6TC1, S1-R3O1-FB, S1-R4D1-FB, and S2-R3O1-FB-BA1 (referred to as the BC isolates, hereafter) could all be identified as *B. cereus* type 9 according to the Sacchi et al. typing scheme. The BA isolates could be identified as type 13 *B. cereus* of the Sacchi et al. scheme, but identification as type 6 *B. anthracis* could not be ruled out due to a W at position 1146 of the latter. Isolate S2-R4W1-FB-BA1 (referred to as the BW isolate, hereafter) could not be designated according to the Sacchi et al. scheme, but had 100% similarity with the Genbank sequences of the type strains *B. mycoides* ATCC 6462^T^ (AB021192) and *B. weihenstephanensis* DSM 11821^T^ (AB021199).

**Table 3:** 16S rDNA sequence signature comparison of isolates according to the Sacchi et[31] type scheme.

16S type	Strain	Nucleotide position												
		77	90	92	182	189	192	200	208	1015	1036	1045	1146	1462
type 9 *B. cereus*		A	T	T	C	A	C	T	G	A	T	A	A	T
	S1-R1P1-FB	A	T	T	C (t)	A	C	T	G	A (c)	T	A	A	T (a)
	S1-R6TC1	A	T	T	C (t)	A	C	T	G	A (c)	T	A	A	T (a)
	S1-R3O1-FB	A	T	T	C (t)	A	C (t)	T	G	A (c)	T	A	A	T (a)
	S1-R4D1-FB	A	T	T	C (t)	A	C (t)	T	G	A (c)	T	A	A	T (a)
	S2-R3O1-FB-BA1	A (g)	T	T	C	A	C (t)	T	G	A (c)	T	A	A	T (a)
type 13 *B. cereus*		A	T	T	C	A	C	T	G	C	T	A	T	T
type 6 *B. anthracis*		A	T	T	C	A	C	T	G	C	T	A	W	T
	S1-R1J2-FB	A	T	T	C (t)	A	C	T	G	C	T	A	T	T
	S1-R2T1-FB	A	T	T	C (t)	A	C	T	G	C	T	A	T	T
	S1-R4H1-FB	A	T	T	C (t)	A	C	T	G	C	T	A	T	T
	S2-R3J1-FB-BA1	A	T	T	C (t)	A	C	T	G	C	T	A	T	T
	S1-R5C1-FB	A	T	T	C (t)	A	C	T	G	C	T	A	T	T
	*B. mycoides* ATCC 6462^T^ (AB021192)	A	T	T	T	A	T	T	G	A	T	A	A	A
	*B. weihenstephanensis* DSM 11821 (AB021199)	A	T	T	T	A	T	T	G	A	T	A	A	A
	S2-R4W1-FB-BA1	A	T	T	T	A	T	T	G	A	T	A	A	A

Abbreviations: R, A or G nucleotide; Y, C or T nucleotide; M, A or C nucleotide; W, A or T nucleotide. Nucleotides between brackets indicate a weak double signal of that nucleotide at that position.

### PCR of *gyrB*

The results of the PCR analysis of *gyrB* are shown in Table 2. The BC isolates were all positive for *B. cereus* PCR, which is in agreement with 16S rDNA sequence analysis. All BA isolates were positive for *B. anthracis* PCR. The BW isolate was positive for *B. anthracis* PCR in two of four reactions.

### Expert Confirmation Analysis

All isolates were determined to be negative for *B. anthracis* by the reference laboratory. Isolate S1-R5C1-FB was slightly inhibited by the phage. The image in a Gram-stained preparation was found to be divergent from *B. anthracis* for all isolates. PCR results for the *B. anthracis* chromosomal marker BA_5345 and the *lef* and *capC* markers on plasmids pXO1 and pXO2, respectively, were negative for all strains.

### Biochemical and Phenotypic Characterisation

All strains were Gram-positive rods with ellipsoidal to cylindrical endospores not swelling the sporangia that lie central to subterminal, which mostly were capable to form chains of cells. All strains were positive for anaerobic growth, starch hydrolysis and lecithinase; all strains were negative for parasporal crystals, growth at 50°C and 65°C, lipase and gas from glucose. In the API 20 E strip, for all strains, nitrate reduction and gelatinase are positive; the indole production test is negative. In the API 50 CHB gallery, acid is produced from glycogen and D-trehalose; acid is not produced from D-arabinose, inulin, and D-mannitol. Of the VITEK 2 Systems BCL card tests, all strains were positive for phenylalanine arylamidase, L-pyrrolydonyl-arylamidase, β-N-acetyl-glucosaminidase, D-glucose, D-ribose, maltotriose and esculin hydrolysis; all strains were negative for putrescine assimilation, methyl-A-D-glucopyranoside acidification, D-galactose, D-melezitose, methyl-D-xyloside, myo-inositol, palatinose, L-rhamnose, D-tagatose, Ellman, ala-phe-pro arylamidase, L-lysine-arylamidase, L-proline arylamidase, α-galactosidase, β-galactosidase, α-mannosidase, β-mannosidase, β-xylosidase. All strains were resistant to polymixin-B and susceptible to oleandomycin. Biochemical and phenotypic characteristics for differentiating the strains from *B*. *cereus*, *B. anthracis*, *B*. *thuringiensis* and *B*. *mycoides* are presented in Table 2. On the basis of the above characteristics, all strains could be identified as members of the *B. cereus* group [22], [30].

Differentiation by colony morphology was consistent with the results of other methods such as PCR of *gyrB* and sequence analysis of the 16S rRNA gene, except for isolate S1-R5C1-FB. Of the VITEK 2 Systems BCL card, the BA isolates were mostly distinguishable from the BC and BW isolates with tests for alanine arylamidase and β-glucosidase. None of the strains tested positive for the *B. anthracis* virulence factor of capsule production.

## Discussion

Naturally occurring anthrax caused by *B. anthracis* is readily controllable, however artificially massive exposures can be created, making it much less controllable and placing it high on the list of potential agents of biological warfare or bioterrorism [5], [22]. As a result, *B. anthracis* has been researched and developed as a biological weapon over many years.

Due to their resistance to desiccation, heat, radiation and disinfectants, spores of aerobic endospore formers readily survive distribution in soils, dusts, and aerosols, making them troublesome contaminants. *B. anthracis* probably only rarely multiplies in the environment [22], [28]. In certain environments however, such as in advanced life support systems like the ISS, build-up of spores of adverse microbial species might occur due to factors such as dust accumulation. In addition, hygiene regimes [27] that make use of cleaning solutions containing disinfectants may clean selectively, favouring microbes and spores that possess features to withstand hostile environments. Indeed, spore-forming bacterial species such as of the genus *Bacillus* have been detected on the ISS in considerable numbers [24].

We isolated strains of members of the *B. cereus* group from the interior of the ISS, of which BC isolates were found to be most similar to *B. cereus* and BW isolates to *B. weihenstephanensis* or *B. mycoides*.

The correct identification of virulent *B. anthracis* is a well-documented challenge [16], [29]. The BA isolates possessed *B. anthracis*-like biochemical characteristics such as acid from glycerol and salicin reactions. Several of the differentiating characters for *B. anthracis* such as a lack of β-haemolytic activity, lack of motility, penicillin sensitivity, susceptibility to γ-phage lysis or some genetic markers are not truly specific [5], [22], [25], [32], making correct identification challenging and thus leaving these characters inconclusive for the BA isolates. Traits as growth on Phenylethyl Alcohol Blood Agar (PEA) and arginine dihydrolase argued against identification of the BA isolates as *B. anthracis*.

Even though the differentiation of *B. anthracis* from *B. cereus* on the basis of 16S rRNA gene sequencing has been reported to be difficult, it has been proven useful for fast differentiation [2], [31]. 16S rRNA gene sequence analysis showed signatures of the BA isolates to be similar to type 13 *B. cereus* and type 6 *B. anthracis* according to the scheme of Sacchi et al. [31]. A conclusive identification of the BA isolates as type 6 *B. anthracis* could however not be made due to a mixed basepair at position 1146 of the latter, which could not be confirmed by the sequencing approach used in our study.

The *gyrB* gene has been proven to be a useful phylogenetic discriminator for members of the *Bacillus cereus* group [19]. Whilst phenotypic characters and 16S rDNA analysis of the BA isolates pointed towards suspect for *B. anthracis* identification, PCR of chromosomal marker *gyrB* of the isolates was positive for *B. anthracis*, however, in contrast, PCR of chromosomal marker BA_5345 was negative for *B. anthracis*.

Regarding the phenotypic and genetic characteristics of the BA isolates and the observation that virulence genes of the pXO1 and pXO2 plasmids were not detectable by PCR, the latter which was confirmed by the lack of a detectable capsule, these isolates may best be defined as non-virulent *B. cereus*/*B. anthracis* sensu lato [25].

Identification of virulent *B. anthracis* may be troublesome for several reasons, in particular in determining the presence of the anthrax virulence plasmids [25]. First of all, merely the possession of both plasmids pXO1 and pXO2 is not enough for *B. anthracis* to cause anthrax. The Carbosap strain of *B. anthracis* used in Italy to vaccinate cattle possesses both plasmids pXO1 and pXO2, but is not lethal, possibly due to low expression [6]. Furthermore, *B. anthracis* isolates may lack one or both of the virulence plasmids [6], [32] and vice versa the virulence plasmids or homologues thereof can occur in close relatives such as *B. cereus*, which may or may not carry functional anthrax virulence genes which can also cause serious disease in humans [11], [13], [17], [20], [25], [28].

Molecular and antigenic methods have been developed for the rapid detection of *B. anthracis* [5], [16], [22], [29]. For the correct identification of virulent *B. anthracis* by molecular assays it is essential that both multiple chromosomal markers such as *rpoB*, *Ba813* or *gyrB* as well as the virulence genes on both plasmids are targeted [12], [29]. In addition to PCR techniques and antigenic assays, there is an increase in reports using other technologies [22], [29]. Most of these methods have been designed to occur after infection is suspected [5] and as such are not relevant for rapid environmental pathogen detection [29]. However, all of these methods rely on the identification of separate markers of virulent *B. anthracis* strains, as a result of which they cannot distinguish between the presence of all markers within the same strain or their presence in the microbial community as a whole and as such, hindering the inconclusive determination of whether or not a virulent *B. anthracis* is detected in a sample of mixed species.

For the detection of virulent *B. anthracis* rapid molecular methods are preferred, however in certain situations this can be problematic. We observed the considerable challenge for rapid molecular on-site detection of virulent *B. anthracis* in the situation that multiple species of the *B. cereus* group are present within the same microbial sample. There is an increased chance for this occurrence in the situation that *B. anthracis*-like bacteria are present in the same environment as other members of the *B. cereus* group. This study describes the preliminary classification of *B. anthracis*-like bacteria which were found to be present on board of the ISS within the same human environment as other members of the *B. cereus* group: *B. cereus*, *B. weihenstephanensis* and *B. mycoides*.

As such this study shows that, besides the known challenges for correctly identifying virulent *B. anthracis* in situations such as of potential biological terrorism, there is also a challenge for monitoring the microbial hygiene in specific human environments. For the monitoring and control of microbial hygiene in human environments at remote locations such as the ISS, it may be difficult to predict certain situations that are critical to human health direct and on-site with a rapid and easy to use molecular methodology, before the emergence of a clinical case. In microbial communities of such environments both *B. anthracis*-like bacteria and other members of the *B. cereus* group may co-exist, as was shown in this study, which is consistent with findings in the natural environment such as soil or aerosols [15], [18], [23]. As such, the two plasmids pXO1 and pXO2 or their homologues, harbouring the virulence factors for anthrax, might occur simultaneously in separate strains of members of the *B. cereus* group within the microbial population, hampering detection of virulent *B. anthracis* by molecular methods. As a result, the risk assessment of the microbial hygiene situation and whether it is becoming dangerous to human health is difficult to determine for this species by the present methods. Our research shows that not only the primary issue of correct identification of virulent *B. anthracis* is important for future research but that it should also focus on finding a solution for the issue of false positives caused by the need to detect multiple markers within the same sample.
